# Unlocking the potential for digital mental health technologies in the UK: a Delphi exercise

**DOI:** 10.1192/bjo.2019.95

**Published:** 2020-01-28

**Authors:** Clare Murphy, Lucy Thorpe, Harriet Trefusis, Antonis Kousoulis

**Affiliations:** Digital Research and Policy Officer, The Mental Health Foundation, UK; Head of Policy, The Mental Health Foundation, UK; Digital Research and Policy Assistant, The Mental Health Foundation, UK; Director of England and Wales, Development and Delivery, The Mental Health Foundation, UK

**Keywords:** Cognitive behavioural therapies, complimentary therapies, information technologies, outpatient treatment, primary care

## Abstract

**Background:**

Digitally enabled services can contribute to the support, treatment and prevention of mental health difficulties; however, questions remain regarding how we can most usefully harness such technology in primary and secondary mental healthcare settings.

**Aims:**

To identify barriers and facilitators to enable the potential of digital mental health in England, Scotland, Wales and Northern Ireland.

**Method:**

A three-round Delphi exercise was carried out online with 16 participants from across the four nations of the UK representing the following stakeholder groups: service providers, health professionals, policymakers, lived experience, small and medium enterprises and academics. Qualitative data were collected in the first round (80 fragments) that were then coded to produce a 26-item round-two questionnaire for participant rating. Participants were given the opportunity to reconsider their scores in light of the group responses in round three.

**Results:**

Eight statements under the following five themes reached consensus with median scores between 8 and 10 (i.e. important/very important): co-production; the human element; data security; funding; and regulation.

**Conclusions:**

The Delphi process allowed consensus to be achieved regarding the factors that experts consider important for harnessing technology in primary and secondary mental healthcare. Knowledge of these factors can help users and providers of mental health services negotiate how best to move forward with digitally enabled systems of care.

With increasing pressure on healthcare budgets across the globe, strategies to improve access to mental healthcare at lower costs are needed.^1^ One such approach is the offer of internet-based or smartphone-based interventions within healthcare services.^1–4^ In the UK, their potential is recognised in health policy documents such as England's National Health Service (NHS) Long-Term Plan (https://www.longtermplan.nhs.uk/). The plan outlines an intention to work with the voluntary sector, developers and individuals in developing a range of apps to support mental health, with an aim to offer digitally enabled models of therapy for anxiety and depression across services by 2020. However, to date, their implementation into public and private mental healthcare services in the UK has been relatively slow. Several factors remain to be considered if we are to harness such technology effectively in primary and secondary mental healthcare, as well as promote its more general use in community settings.^5,6^ Given the relative infancy of digitally enabled healthcare services in the UK, there is still much to learn about the best ways to implement and utilise digital services in mental healthcare.^7^

The aim of the current study is to achieve expert consensus on the barriers and facilitators to unlocking the potential of digital mental health in the four nations of the UK to guide future areas of research and policy action. As this is a relatively new research domain, where previous longitudinal studies or systematic research is sparse, the Delphi process was considered appropriate to gauge current expert opinion.^8^

## Method

The Delphi process is an approach that utilises both qualitative and quantitative methods to establish a consensus opinion on issues of identified importance.^9,10^ The technique has been used in a variety of fields since its development in the 1950s^11^ and uses structured group communication to facilitate individual assessment of group judgement, allowing for revision of opinions, while protecting participant anonymity.^12^ This Delphi exercise was conducted by researchers at the Mental Health Foundation (London, UK) between 19 July and 6 September 2018. Ethical approval for this study was obtained by Queens University Belfast (11 April 2018). Written informed consent was obtained from all participants prior to execution.

### Participants

Using a non-random purposive sampling technique, participants were selected based on their expert knowledge of digital mental health in the UK. To form a representative panel, experts based in the four nations in the UK, from the following six stakeholder groups were invited to participate: service providers; health professionals; policymakers; people with lived experience; small and medium enterprises, and academics. Participants were identified through horizon scanning of the field of digital mental health, and a review of UK-based literature published between 2016 and 2018. A matrix of 30 participants who met our inclusion criteria was created. Participants were categorised into the stakeholder group that they contributed the most to within the previous 5 years (2013–2018). Of the 30 participants selected, 25 were contactable. One participant from each of the six stakeholder groups was invited (by email) from each of the four countries (two participants for the stakeholder category ‘service providers’ in England were contacted) (*n* = 25). Care was taken to protect the anonymity of those contacted. Twenty-one agreed to take part and were sent a consent form to return. Of those, 19 signed and returned the consent form. The final sample consisted of 16 participants (10 men; 6 women) from the four countries of the UK (England (*n* = 6); Northern Ireland (*n* = 5); Scotland (*n* = 3); and Wales (*n* = 2)). The number of participants represented in each stakeholder group is shown in Table 1.

**Table 1:** Final sample of participants in each stakeholder group and country (each participant that took part represented one stakeholder group for one country, and no more than that)

Stakeholder group	England, *n*	Scotland, *n*	Wales, *n*	Northern Ireland, *n*	Total, *n*
Service providers (i.e. mental health charities and public healthcare providers)	2	1	0	1	4
Health professionals (i.e. psychiatry)	1	0	0	1	2
Policymakers	1	1	1	0	3
Lived experience	1	0	0	1	2
Small and medium enterprises (i.e. app developers)	0	1	0	1	2
Academics (i.e. university professor)	1	0	1	1	3
Total	6	3	2	5	16

### Round one

For round one all participants were directed to a survey hosted at MySurveyLab™ which gave them the following instruction: ‘Please give five different examples of what, in your opinion, it would take to unlock the potential of digital tools for mental health in the UK. It would be helpful in your responses to bear in mind the potential facilitators/barriers to the implementation of e-mental health in the UK. Please do not exceed 100 words per example.’ Responses were recorded via free-text boxes. Non-responsive participants were contacted with reminder emails by the lead researcher.

To construct the survey for round two, open coding was used (see Data analysis).

### Round two

Round two of the Delphi process was also hosted at MySurveyLab™. Participants were asked to rate the statements generated from round one on a ten-point Likert scale (ranging from 1 ‘not important’ to 10 ‘very important’) regarding their ‘importance for unlocking the potential of digital tools for mental health in the UK’. Round two was open for 3 weeks. The data-set was exported and analysed using Microsoft Excel software.

### Round three

**Fig. 1:**
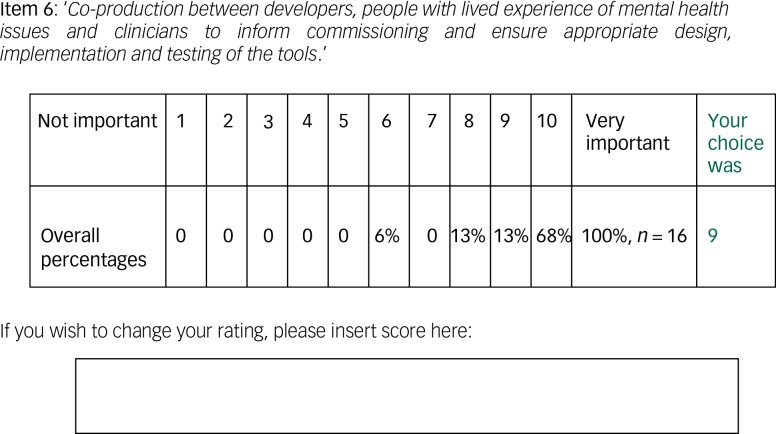
Example of round-three feedback with group-percentage scores and individual's score for each item.

In round three, participants were invited to reconsider their round-two scores in light of the group responses. The round-three survey contained the same items from round two, with each item accompanied by a table to illustrate group-percentage scores as well as a reminder of their individual score (Fig. 1). This provided each participant with a quick visual means of assessing the diversity of responses.^8^ Round three was closed within 3 weeks.

Six participants (47%) made changes to their original round-two scores of between 1 and 3 points, one participant made one change of 4 and of 5 points to two items. The data were re-analysed in lieu of the new scores. Percentages, medians and interquartile ranges (IQRs) were calculated. Table 2 shows the median scores and IQRs for the 26 items in round three of the data collection.

**Table 2:** Medians and interquartile ranges (IQRs) calculated for round three of the data collection

Item	Median	IQR
Item 1	5	4.25
Item 2	7.5	4.25
Item 3	9.5	3
Item 4^a^	5	1.75
Item 5	7.5	2.5
Item 6^a^	10	0.25
Item 7^a^	10	1
Item 8	9.5	3
Item 9^a^	10	1.25
Item 10	9.5	5
Item 11	9	2.25
Item 12^a^	9	2
Item 13^a^	8	2
Item 14	8	3.5
Item 15	9	3.25
Item 16	9	2.25
Item 17	9	2.25
Item 18^a^	9	2
Item 19	8	3.25
Item 20	8	3.25
Item 21	7	3.25
Item 22	8.5	2.5
Item 23	8	2.25
Item 24^a^	8	1.5
Item 25^a^	8	1
Item 26	8	2.25

a. Items with a median ≥ 5 and an IQR between 0 and 2.

### Data analysis

A total of 80 statements were generated by 16 participants for round one. Open coding was used to establish themes running through the data. Each statement was read and re-read by the C.M. and H.J. separately and the themes identified were given codes. They then organised their results into a matrix data-set, wherein each coded theme was presented alongside supporting statement(s). After this, the authors compared, collated and discussed their matrices until agreement was reached on the meaning of each item and its related theme. This was a dynamic, iterative process of organising and synthesising participants’ responses, wherein long responses were summarised, exact duplicates were removed and broadly similar responses were amalgamated. This process was overseen by a third, independent researcher to produce the final survey for round two.

For analysis of round-two responses, basic statistics were generated by MySurveyLab™, which were exported to Excel to calculate the medians and IQR of scores participants gave for each statement in round two and round three. A pre-agreed consensus of an IQR no larger than 2 units on a 10-unit scale was applied.^13–15^

## Results

In terms of the response rate for this survey, each round closed within approximately 3 weeks, with a response rate of 100% for round one and two and 93% in round three. For round one, a total of 80 responses were submitted by 16 participants. Open coding produced a final list of 26 easy-to-understand, one to two line statements, which were used for the round-two survey (see supplementary File 1 available at https://doi.org/10.1192/bjo.2019.95). These statements were kept as close to the participants’ original statements as possible. The results of the round indicated 8 of the 26 statements (31%) reached consensus (IQRs 0.25 to 2) with median scores between 8 and 10. Statements with lower median scores are not presented here. These eight statements were categorised under the following five themes: (a) co-production (one item); (b) data security (two items); (c) the human element (two items); (d) funding (one item); and (e) regulation (two items) (see Table 3).

**Table 3:** Eight items that reached consensus and their overarching themes

Theme, item number	Item	Median (IQR)
Co-production		10 (0.25)
Item 6	Co-production between developers, people with lived experience of mental health issues and clinicians to inform commissioning and ensure appropriate design, implementation and testing of the tools.	
Data security		
Item 7	Have sufficiently robust information governance structure and clarity about issues related to collecting patient data, confidentiality, storage of data, access to data etc.	10 (1)
Item 9	Trust and accountability in relation to the organisations that produce the tools and transparency about whether data are used for other purposes, such as commercial benefit.	10 (1.25)
The human element		
Item 24	Develop tools that facilitate two-way communication between clinicians and users.	8 (1.5)
Item 25	Develop e-mental health products that include facilities for support and mentoring.	8 (1)
Funding		
Item 18	Finance available for adoption, testing and implementation of digital tools.	9 (2)
Regulation		
Item 12	Clear regulatory framework of standards in relation to the risk, safety, effectiveness and reliability of digital tools, with quality criteria and evidence base for digital tools.	9 (2)
Item 13	Increased understanding in the public sector to make informed decisions on where technology is useful, in the patient pathway, particularly when commissioning.	8 (2)

IQR, interquartile range.

## Discussion

This Delphi process allowed consensus to be achieved regarding key factors that experts consider important for harnessing technology in primary and secondary mental healthcare.

### Theme 1: co-production

With growing public interest in digital mental health, there is a need for key stakeholders to come together and consider how best to implement digital technologies in a way that is suitable to all strands of the population. Indeed, according to Hill *et al*,^5^ collaboration between clinicians, researchers, industry and users could help navigate the challenges in developing digital mental health innovations and ensure that they are engaging, acceptable, evidence based, scalable and sustainable. Stakeholders should share and disseminate information, expertise and experience through collaborative online platforms, which can be easily facilitated by technology itself. Virtual meeting spaces and platforms, including the International Society for Research on Internet Interventions, offer ideal opportunities for learning from international colleagues, not only in terms of developing and evaluating digital tools, but also in terms of the long-term planning for how innovations can be integrated into existing care pathways and be of practical benefit to users and providers. Such collaboration will help ensure that the online solution matches the needs of the service and its care pathways and could include external agencies, such as the UK's Academic Health Science Networks (AHSN) for guidance on development and adoption by service providers.^5^ Collaboration is needed to support commissioners and decision-makers in the area of digital mental health, and research (particularly the specification of research questions in this area) must involve the people affected by such decisions.^6^ Finally, co-production is also important where children and young people are concerned, as recent research shows that differences exist between what young people search for, find, and prefer to interact with online, and the sources that clinicians would consider authoritative.^16^ Co-production will increase the chances that young people will find the mental health information and the support that they want and need.^17^

### Theme 2: data security

The lack of clear and consistently applied regulations around mental health apps leaves room for improvement for product developers, who will require support and guidance regarding how to assuage uncertainty on the part of users.^18^ Various regulatory frameworks, such as the newly implemented General Data Protection Regulation, are emerging to help inform decision-making for users and to support developers in reaching the level of transparency that users require.^19^ Such standards and regulations can help guide clinicians and users to the most effective tools as well as avoid those that may be less safe. It also obliges both private and public organisations that handle sensitive data to provide the public with the information about the type of data collected and for what reason. Finally, this commits organisations to store such data safely and securely. Further research on the safety measures that should be taken in data protection, privacy and security is needed in order to maintain public and patient trust in the area of digital mental health.

### Theme 3: the human element

A recent systematic review of digital health interventions for children and young people with mental health difficulties indicated that human support is an important factor in influencing uptake, engagement and outcomes.^20^ The type and extent of the human support needed when using online tools will differ from person to person and requires further study. It is also likely that the features needed to support engagement will differ significantly across populations, age groups and mental health conditions. Nevertheless, mental health professionals emphasise the importance of digital tools being considered as adjuncts to traditional face-to-face therapies, rather than a replacement for them.^20^ Research could explore whether digital mental health products that include human support within their design and/or are delivered within the context of blended care achieve better engagement, treatment adherence and outcome. Indeed, recent research indicates that questions around the effect of digital interventions on therapeutic alliance, and concerns about removing face-to-face treatments, were raised by people with lived experience of mental health problems, their carers and healthcare and social care practitioners.^6^

### Theme 4: funding

As a relatively new field, digital mental health is met with limited funding options. Successful large-scale adoption and dissemination of digital mental health interventions in the UK will likely need support of the NHS. Product developers could consider how their tool meets the demands of services as well as existing care pathways. By understanding this market, they can outline clear plans regarding how their product will benefit individuals who use them, clinicians and service providers, along with a strategy for implementation.^5^ NHS England-funded AHSNs and the National Institute for Health Research MindTech MedTech Co-operative (https://www.mindtech.org.uk/), which support the development and evaluation of healthcare innovations, can also help facilitate more swift adoption of verified products and services into the NHS.^5^

In academia, funding is typically slow to secure and is unlikely to cover any ongoing phase of technical development or online maintenance after the end of the funding period. Disseminating products that can generate revenue to cover these costs would be sensible. The lengthy process of evidence-based research is likely to be unappealing for developers, reluctant to invest the time needed for ‘gold standard’ evaluation, such as randomised controlled trials. Methods of evaluating products more efficiently are needed.^21^ More generally, funding should be allocated to the accessibility of digital tools. It is well documented that rural communities are disadvantaged in terms of scope and speed of access to mental healthcare in comparison with urban areas. One of the benefits of digital mental health is its far-reaching potential, offering access to people in any part of the UK. However, the availability of such technology is sometimes undermined by network infrastructure that is slow, unreliable even or absent.^22,23^ Funding should be made available to ensure that mental health services are accessible to the whole population, with a particular focus on underserved groups.

### Theme 5: regulation

One of the challenges associated with integrating digital into mental health services includes the lengthy process of validating and establishing a strong evidence base for the intervention, such that it is suitable for clinical settings. Indeed, the speed with which technology evolves, compared with the slow pace of research, means that the technology could be outdated by the time the intervention is validated.^24^ Moreover, where clinical and research communities have been comparably slow to take up the digital mental health agenda as a priority, commercial industries have seized its lucrative potential, virtually saturating the commercial marketplace with unregulated mental health-related apps and support tools, making it difficult for users and clinicians to identify which ones are effective, safe or beneficial.^19^ Moving forward, this will likely be better managed by introducing regulatory frameworks, which can facilitate informed decision-making around online support tools. Nevertheless, in a time when digital products can reach the marketplace before any evaluation of their efficacy,^5^ great care must be taken that policy and practice around digital mental healthcare does not outpace evidence-based research and risk losing public confidence.^6^

### Limitations

Although participants (*n* = 24) from six stakeholder groups (from all four countries of the UK) were invited to take part in this study, only 16 remained in the final sample, with England and Northern Ireland having the highest representation and Scotland and Wales having the lowest. It is possible that some participants did not respond because of busy schedules and the time required to complete all three rounds. In the final sample, only two individuals with lived experience participated. These participants also work in the field of mental health advocacy and thus may have a vested interest in the field. Future research should allocate more time to replace participants who have not responded and ensure that the final sample adequately represents the views of stakeholder groups from the four nations. In the data-analytic method used in this study statistical interrater reliability was not calculated. Future studies could include this to increase the reliability of the findings. Finally, as experts were consulted for this study, the sample used will not represent the full spectrum of perspectives in digital mental health, such as members of the public.

### Implications

In conclusions, determining the role that technology can play in improving receipt and provision of care could comprise a step towards bringing the NHS back to its seminal promise of equal access for equal need.^25^ The present Delphi study allowed consensus to be achieved between experts on the factors considered to be important for unlocking the potential of digital tools for mental health in the UK, particularly with respect to possible facilitators/barriers to its implementation into services. The factors highlighted here include co-production, data security, the human element, funding and regulation. A focus on these areas can help users and providers of mental health services negotiate how best to move forward with digitally enabled systems of care.

## Data Availability

All authors have full access to the study data.

## Supplementary material

For supplementary material accompanying this paper visit http://dx.doi.org/10.1192/bjo.2019.95. click here to view supplementary material
