# Supplementary material for: Unlocking the potential for digital mental health technologies in the UK: a Delphi exercise
Source: BJPsych Open. 2020 Jan 28;6(1):e12. doi: 10.1192/bjo.2019.95 (PMC7001470; doi:10.1192/bjo.2019.95)
Supplement: Supplementary file 1 [file S2056472419000954sup001.docx]

Appendix 1: Final of 26 statements used for the round two survey

| 1. Integrate services by linking NHS trusts and the voluntary sector within mental health delivery frameworks for referral and information sharing. 2. Create a universal electronic documentation system, which is interoperable across trusts and IT systems. 3. A patient portal to enable patients and carers to access their information and communicate with their care providers. 4. Have a decentralised approach, where the government and public sector have supporting roles in the commissioning and procurement of digital tools, but not a leading role. 5. Flexible tools which are designed specifically for individuals and their changing mental health needs rather than offering universal mental health interventions. 6. Co-production between developers, people with lived experience of mental health issues and clinicians to inform commissioning and ensure appropriate design, implementation and testing of the tools. 7. Have sufficiently robust information governance structure and clarity about issues related to collecting patient data, confidentiality, storage of data, access to data etc. 8. Place the citizen at the centre of their data, which is available to them at any time. 9. Trust and accountability in relation to the organisations that produce the tools and transparency about whether data is used for other purposes, such as commercial benefit. 10. Scientific evidence based and evaluated products to ensure confident use and effectiveness of the tool compared with other interventions. 11. Streamlined research and piloting process to ensure ethical approval is granted quickly and clinicians can easily implement tools. 12. Clear regulatory framework of standards in relation to the risk, safety, effectiveness, and reliability of digital tools, with quality criteria and evidence base for digital tools. 13. Increased understanding in the public sector to make informed decisions on where technology is useful, in the patient pathway, particularly when commissioning. 14. The technology and IT equipment should meet clinicians’ needs with, for example, handwriting recognition and speech recognition for efficient note-taking. 15. Technology should clearly fit into the patient pathway to either enhance experience or unlock efficiencies. 16. Flexible hardware / digital equipment (familiar/usable) that can be used while on the go. 17. General Infrastructure, particularly in terms of internet coverage in rural areas. 18. Finance available for adoption, testing and implementation of digital tools. 19. Clear plans for future development and implementation of digital tools, particularly in terms of horizon scanning and market analysis. 20. More technological skills and understanding of the value of e-mental health in the NHS workforce and culture facilitated by staff training. 21. Widespread digital literacy needed so that users can develop technical skills to understand how to use digital tools. 22. User friendly interface and design; an interface that is customisable for each individual user/clinician. 23. Availability of tools in different languages and communication methods. 24. Develop tools that facilitate two-way communication between clinicians and users. 25. Develop e-mental health products that include facilities for support and mentoring. 26. Develop applications that facilitate peer support. |
| --- |
